# Suppressed Akt/GSK-3β/β-catenin signaling contributes to excessive adipogenesis of fibro-adipogenic progenitors after rotator cuff tears

**DOI:** 10.1038/s41420-023-01618-4

**Published:** 2023-08-25

**Authors:** Xingzuan Lin, Peng Wang, Wei Wang, Hao Zhou, Siyuan Zhu, Shujing Feng, Yuzhou Chen, Han Zhou, Qichao Wang, Hanlong Xin, Xiexiang Shao, Jianhua Wang

**Affiliations:** 1 Xinhua Hospital Affiliated to Shanghai Jiao Tong University School of Medicine, Shanghai, China; 2 Hubei Provincial Hospital of Traditional Chinese Medicine, Wuhan, China; 3 Shanghai University of Sport, Shanghai, China; 4 Taizhou Hospital of Zhejiang Province Affiliated to Wenzhou Medical University, Taizhou, China

**Keywords:** Stem cells, Diseases

## Abstract

Muscular fatty infiltration is a common and troublesome pathology after rotator cuff tears (RCT), which mainly derives from fibro-adipogenic progenitors (FAPs). Compared to the RCT, fatty infiltration is not so severe in Achilles tendon tears (ATT). The knowledge of why fatty infiltration is more likely to occur after RCT is limited. In this study, more severe fatty infiltration was verified in supraspinatus than gastrocnemius muscles after tendon injury. Additionally, we revealed higher adipogenic differentiation ability of RCT-FAPs in vitro. Activation of Akt significantly stimulated GSK-3β/β-catenin signaling and thus decreased PPARγ expression and adipogenesis of RCT-FAPs, while the inhibition effect was attenuated by β-catenin inhibitor. Furthermore, Wnt signaling activator BML-284 limited adipogenesis of RCT-FAPs, alleviated muscular fatty infiltration, and improved parameters in gait analysis and treadmill test for RCT model. In conclusion, our study demonstrated that suppressed Akt/GSK-3β/β-catenin signaling increased PPARγ expression and thus contributed to excessive adipogenesis in RCT-FAPs. Modulation of Akt/GSK-3β/β-catenin signaling ameliorated excessive fatty infiltration of rotator cuff muscles and improved shoulder function after RCT.

## Introduction

Rotator cuff tears (RCT) are common musculotendinous injuries which lead to functional disability and persistent pain for a large number of patients [1, 2]. Secondary muscle degeneration, including fatty infiltration, is a critical contributor to RCT [3]. Although surgical repair is frequently performed to treat ruptured rotator cuff tendon, it cannot attenuate the fatty infiltration in skeletal muscle [4, 5]. Progressive fatty degeneration significantly harms the elasticity and contraction strength of skeletal muscles, leading to retear of the repaired tendon [1, 4, 6]. Thus, fatty infiltration after RCT plays a significant role in both clinical manifestations before surgery and clinical outcomes after surgery.

Although the detailed mechanism of muscular fatty infiltration after RCT is still unknown, the role of fibro-adipogenic progenitors (FAPs) has always been emphasized. It has been reported that approximately 96% of cells which expressed adipogenic marker adiponectin in supraspinatus muscles were derived from fibro-adipogenic progenitors (FAPs) after tendon injury [7]. Both the number of FAPs and the adipogenic differentiation potential of FAPs increased in large sizes of RCT [8]. Furthermore, previous studies also reported that PDGFRα pathways inhibitor imatinib [1], retinoic receptors agonist adapalene [9], and β-3 agonist amibegron [10] could suppress adipogenic differentiation of FAPs and mitigate fatty infiltration in RCT. Thus, FAPs significantly contribute to ectopic fatty formation in rotator cuff muscles, and it is a promising strategy to ameliorate excessive fatty infiltration by targeting FAPs after RCT.

However, the in-depth and detailed mechanism underlying excessive fatty filtration caused by FAPs after RCT has not been fully clarified. Although AKT signaling and WNT5a/GSK3/β-catenin pathway have been solidly demonstrated as crucial pathways for FAPs adipogenesis [11–13], the roles of these pathways in FAPs after RCT are still waiting to be explored. It is worth noting that muscular fatty infiltration is infrequent and not so severe after other tendon tears, especially for Achilles tendon tears (ATT) [14, 15]. This phenomenon indicates that there might exist distinct differences between FAPs from rotator cuff muscle and gastrocnemius muscle [16, 17]. Clarifying such differential mechanisms might help understand the frequent fatty infiltration after RCT. Thus, the current study was performed to investigate the functional differences between FAPs from RCT and ATT, examine the underlying mechanisms of excessive fatty infiltration after RCT and propose a potential strategy to prevent or abate the progress of fatty infiltration.

Here, we found a greater propensity for adipogenesis of FAPs isolated from RCT compared to ATT. Mechanically, suppressed Akt/GSK-3β/β-catenin pathways increased PPARγ expression, leading to excessive adipogenesis of RCT-FAPs. Activation of Akt/GSK-3β/β-catenin signaling could be a therapeutic strategy to abate excessive fatty infiltration of rotator cuff muscle and improve function of shoulder joint after RCT.

## Results

### There was more fatty infiltration in supraspinatus muscle than gastrocnemius muscle in tendon injury model

**Fig. 1:**
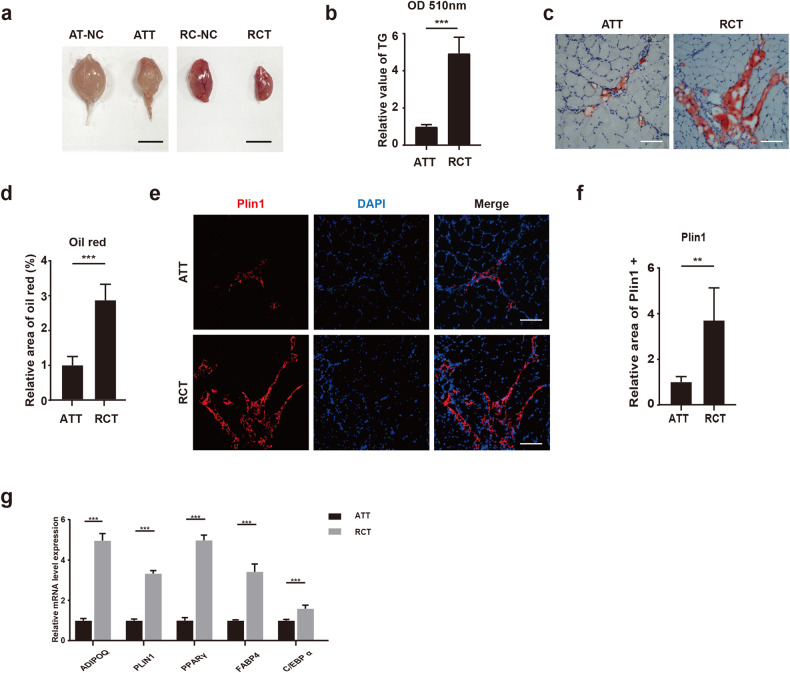
There was more fatty infiltration in supraspinatus muscle than gastrocnemius muscle in tendon injury model. **a** Gross appearance of gastrocnemius and supraspinatus muscles six weeks after tendon injury or without tendon injury. AT-NC Achilles tendon-negative control, ATT Achilles tendon tear, RC-NC rotator cuff-negative control, RCT rotator cuff tear. Scale bar = 50 mm. **b** Measurement of triglycerides in gastrocnemius and supraspinatus muscles after tendon injuries (*n* = 5). **c**, **d** Oil red staining and statistical analysis for gastrocnemius and supraspinatus muscles after tendon injuries (*n* = 5). Scale bar = 100 µm. **e**, **f** Immunofluorescence staining and statistical analysis for gastrocnemius and supraspinatus muscles after tendon injuries (*n* = 5). Scale bar = 100 µm. **g** The relative mRNA expression of adipogenic-related genes in gastrocnemius and supraspinatus muscles after tendon injuries (*n* = 4). ** indicated *p* < 0.01, and *** indicated *p* < 0.001.

The Achilles tendon and rotator cuff injury mouse models were first established by transverse incision of tendon. Six weeks after tendon injury, muscle degeneration was confirmed in supraspinatus and gastrocnemius muscles (Fig. 1a). There were more triglycerides in supraspinatus than gastrocnemius muscle (Fig. 1b). Consistently, more lipids in supraspinatus muscle were verified by oil red staining and perilipin1 immunofluorescence staining (Fig. 1c–f). Furthermore, the expression of adipogenic-related genes was also significantly higher in the supraspinatus muscle than gastrocnemius muscle (Fig. 1g). Taken together, these data demonstrated that fatty infiltration was more severe in supraspinatus muscle than that in gastrocnemius muscle in current tendon injury models.

### There was similar proliferation ability between RCT-FAPs and ATT-FAPs

**Fig. 2:**
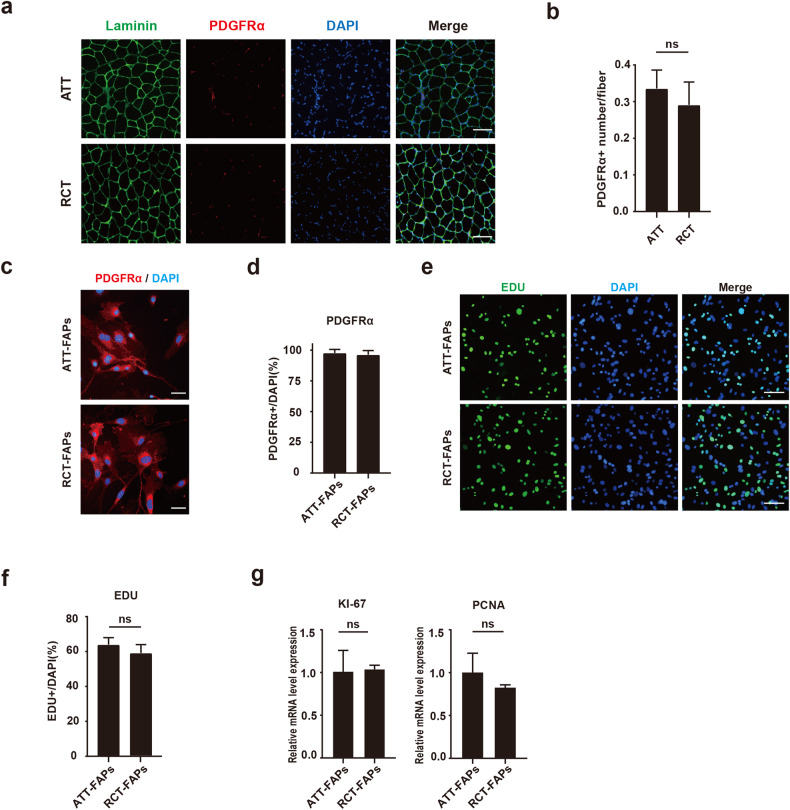
There was similar proliferation ability between RCT-FAPs and ATT-FAPs. **a** Immunofluorescence staining of PDGFRα and laminin for supraspinatus and gastrocnemius muscle one week after tendon injuries (*n* = 5). ATT achilles tendon tear, RCT rotator cuff tear. Scale bar = 100 µm. **b** Statistical analysis of PDGFRα positive cell number. **c** Immunofluorescence staining of PDGFRα for ATT-FAPs and RCT-FAPs (*n* = 6). Scale bar = 50 µm. RCT-FAPs FAPs sorted from supraspinatus and infraspinatus one week after tendon injuries, ATT-FAPs FAPs sorted from gastrocnemius muscle one week after tendon injuries. **d** Statistical analysis of percentage of PDGFRα positive cells. **e**, **f** EDU staining and statistical analysis for RCT-FAPs and ATT-FAPs (*n* = 5). Scale bar = 100 µm. **g** The relative mRNA expression of KI-67 and PCNA between RCT-FAPs and ATT-FAPs (*n* = 3). The ns indicated *p* > 0.05.

We then investigated why more fatty infiltration occurred in supraspinatus muscle after tendon injury. Since FAPs were the primary source of muscular fatty infiltration after tendon injury [7], we focused on the differences of FAPs between supraspinatus and gastrocnemius muscles. One week after tendon injury, the immunofluorescence staining of FAPs marker PDGFRα indicated no significant difference in the density of FAPs in vivo (Fig. 2a, b). Then the FAPs were isolated by fluorescence-activated cell sorting and verified by PDGFRα staining (Fig. 2c, d). The proliferation ability of freshly isolated RCT-FAPs and ATT-FAPs was compared. No statistical significance was found in percentage of EDU positive cells between ATT-FAPs and RCT-FAPs (Fig. 2e, f). Consistently, there was also no significant difference in the mRNA expression of proliferation-related genes KI-67 and PCNA between these cells (Fig. 2g). Thus, these data indicated similar proliferation ability between RCT-FAPs and ATT-FAPs.

### RCT-FAPs showed higher adipogenic differentiation ability than ATT-FAPs

**Fig. 3:**
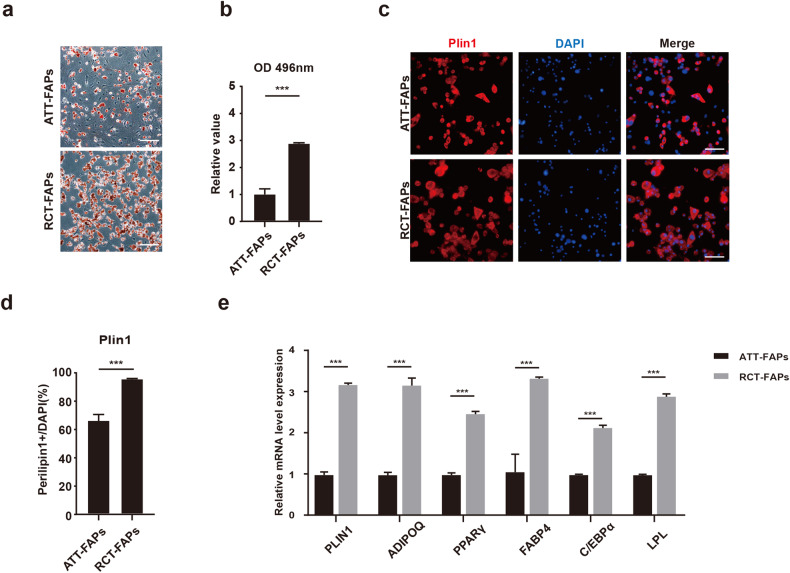
RCT-FAPs showed higher adipogenic differentiation ability than ATT-FAPs. **a**, **b** The oil red staining and statistical analysis for ATT-FAPs and RCT-FAPs after 7-day adipogenic induction (*n* = 3). Scale bar = 100 µm. ATT-FAPs FAPs isolated from gastrocnemius muscle one week after tendon tears, RCT-FAPs FAPs isolated from supraspinatus muscle one week after tendon tears. **c**, **d** Immunofluorescence staining of perilipin1 and statistical analysis for ATT-FAPs and RCT-FAPs after 7-day adipogenic induction (*n* = 3). Scale bar = 50 µm. **e** Relative mRNA expression level of PLIN1, ADIPOQ, PPAR γ, FABP4, C/EBPα, and LPL for ATT-FAPs and RCT-FAPs after adipogenic induction (*n* = 3). *** indicated *p* < 0.001.

We then investigated whether there was a higher adipogenic differentiation potential of RCT-FAPs after tendon injury. After adipogenic induction, more lipid marked by oil red was observed in RCT-FAPs than in ATT-FAPs (Fig. 3a, b). Immunofluorescence staining also revealed a higher percentage of perilipin1 positive cells in RCT-FAPs after adipogenic induction (Fig. 3c, d). Consistently, the mRNA expression of adipogenic genes was also significantly higher in differentiated RCT-FAPs than ATT-FAPs (Fig. 3e). Together, these data demonstrated that there was a higher adipogenic differentiation ability of RCT-FAPs than ATT-FAPs.

### RNA-seq analysis revealed that Akt, Wnt, and PPAR signaling pathways could be key mechanisms for FAP adipogenesis

**Fig. 4:**
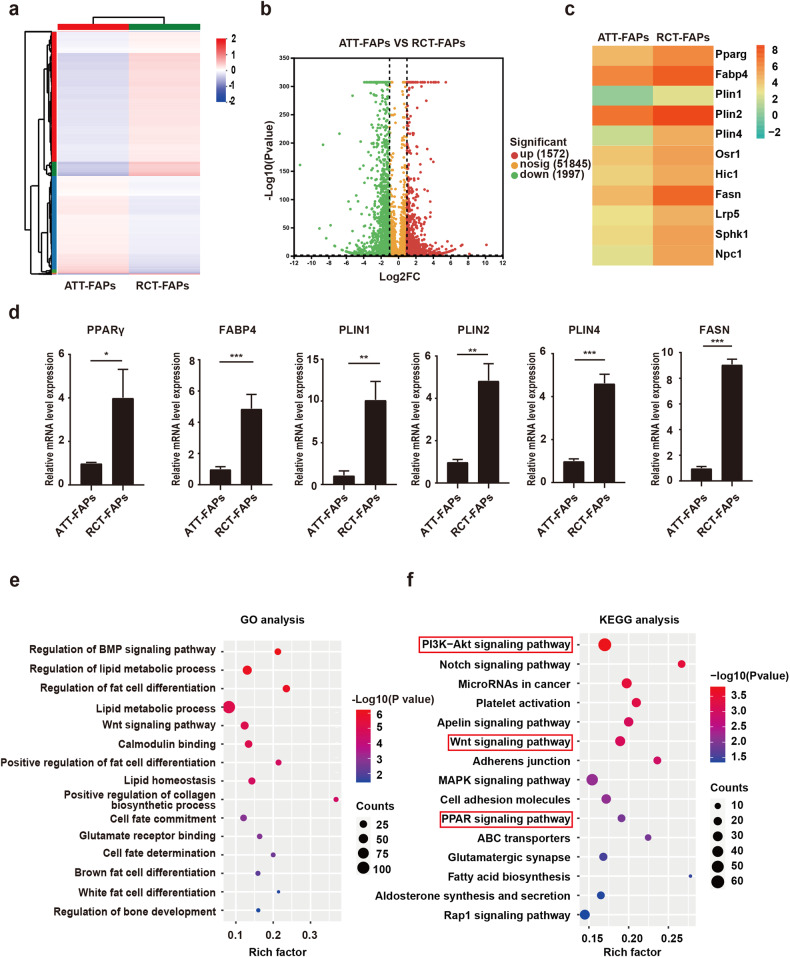
RNA-seq analysis revealed that Akt, Wnt, and PPAR signaling pathways could be key mechanisms for FAP adipogenesis. **a** Heatmap of clustering analysis for differentially expressed genes between ATT-FAPs and RCT-FAPs. ATT-FAPs FAPs collected from gastrocnemius muscle one week after tendon tears, RCT-FAPs FAPs collected from supraspinatus muscle one week after tendon tears. **b** Volcano map for differentially expressed genes between ATT-FAPs and RCT-FAPs. **c** Heatmap of adipogenic-related gene expression profiles of ATT-FAPs and RCT-FAPs. **d** Relative mRNA expression level of adipogenic-related genes for RCT-FAPs and ATT-FAPs (*n* = 3). The * indicated *p* < 0.05, ** indicated *p* < 0.01, and *** indicated *p* < 0.001. **e** Bubble chart of GO analysis of differentially expressed genes. **f** Bubble chart of KEGG analysis of differentially expressed genes.

We next performed the RNA-sequence to compare the expression profile between freshly isolated RCT-FAPs and ATT-FAPs. The differentially expressed gene analysis results were shown by heatmap and volcano chart (Fig. 4a, b). RNA-sequence revealed higher expression of adipogenic-related genes in RCT-FAPs than ATT-FAPs, which was also confirmed by RT-qPCR (Fig. 4c, d). Consistently, GO and KEGG enrichment analysis of differentially expressed genes also enriched fatty infiltration related terms, including fat cell differentiation, lipid metabolic process, and fatty acid biosynthesis (Fig. 4e, f). Thus, these data demonstrated increased transcription of genes involved in adipogenic differentiation tendency of RCT-FAPs.

In addition, it was worth noting that the Wnt signaling pathway, PPAR signaling pathway, and PI3K-Akt signaling pathway were highly enriched by GO and KEGG analysis (Fig. 4e, f). Previous studies reported that Akt/GSK-3β/β-catenin pathway could regulate the adipogenic differentiation of bone marrow mesenchymal stem cells by targeting PPARγ [18, 19]. Since GSK-3β and β-catenin were key effectors in canonical Wnt signaling, the GO and KEGG analysis indicated that Akt, Wnt, and PPAR signaling pathways could be key mechanisms for increased adipogenic differentiation tendency of RCT-FAPs.

### Suppressed Akt/GSK-3β/β-catenin signaling upregulated PPARγ expression and increased adipogenic differentiation potential of RCT-FAPs

**Fig. 5:**
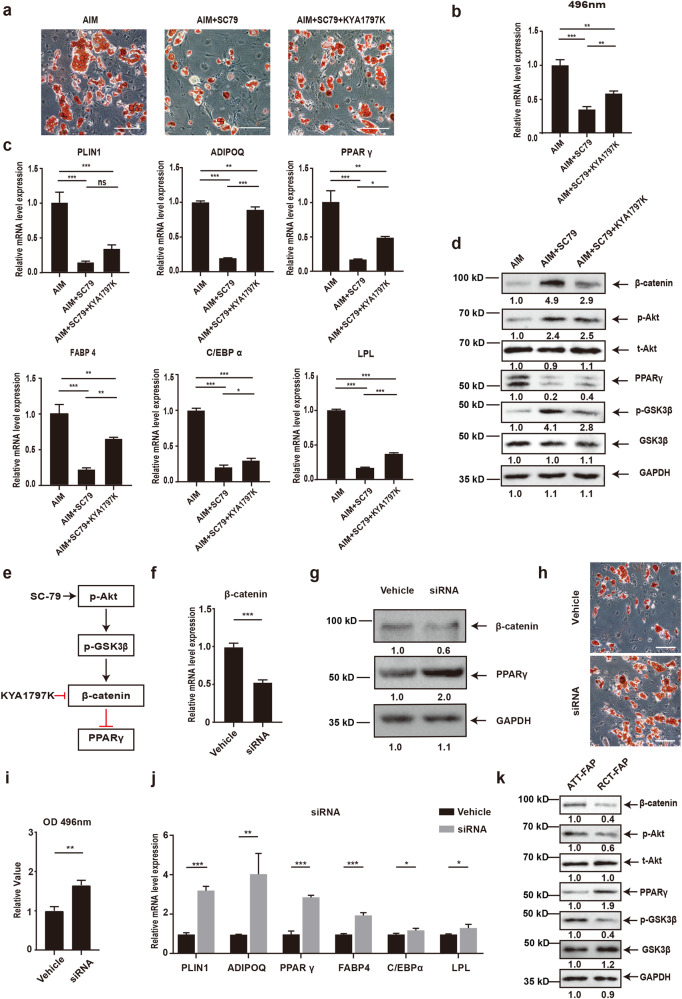
Suppressed Akt/GSK-3β/β-catenin signaling upregulated PPARγ expression and increased adipogenic differentiation potential of RCT-FAPs. **a**, **b** The oil red staining and statistical analysis of RCT-FAPs (FAPs collected from supraspinatus muscle one week after tendon tears) with different treatments. Freshly isolated RCT-FAPs were cultured in AIM, AIM with Akt agonist SC-79, AIM with SC-79 and β-catenin inhibitor KYA1797K for 7 days (*n* = 3). Scale bar = 100 µm. AIM adipogenic induction medium. SC-79, Akt signaling agonist; KY1797K, β-catenin inhibitor. **c** Relative mRNA expression level of adipogenic-related genes for RCT-FAPs after adipogenic differentiation with different treatments (*n* = 3). **d** Protein level of β-catenin, p-Akt, t-Akt, PPARγ, p-GSK-3β, GSK-3β, and GAPDH for RCT-FAPs treated by AIM, AIM with SC-79, AIM with SC-79 and KYA1797K. **e** The scheme of Akt/GSK3β/β-catenin signaling, which regulated adipogenesis of FAPs. **f** Relative mRNA expression level of β-catenin after transfected with β-catenin siRNA in RCT-FAPs (*n* = 3). **g** The protein levels of β-catenin and PPARγ after transfected with β-catenin siRNA in RCT-FAPs (*n* = 3). **h**, **i** The oil red staining and statistical analysis of RCT-FAPs after β-catenin siRNA transfection and adipogenic induction (*n* = 3). Scale bar = 100 µm. **j** Relative mRNA expression level of adipogenic genes for RCT-FAPs after β-catenin siRNA transfection and adipogenic induction. (*n* = 3). **k** Protein level of β-catenin, p-Akt, t-Akt, PPAR γ, p-GSK-3β, GSK-3β, and GAPDH for RCT-FAPs and ATT-FAPs. * indicated *p* < 0.05, ** indicated *p* < 0.01, and *** indicated *p* < 0.001.

Next, we explored whether adipogenic differentiation of RCT-FAPs was regulated by Akt/GSK-3β/β-catenin pathway. SC-79 is a known Akt activator. Freshly isolated RCT-FAPs were cultured in AIM with or without SC-79 for 7 days. Oil red staining and RT-qPCR showed that activation of Akt significantly decreased the adipogenic differentiation ability of RCT-FAPs (Fig. 5a–c). Furthermore, when the β-catenin inhibitor KYA1797K was added in AIM with SC-79, the inhibitory effect of adipogenic differentiation by SC-79 was attenuated (Fig. 5a–c). Western blotting results showed that activation of Akt signaling increased phosphorylated levels of GSK-3β and β-catenin while reduced expression of PPARγ. In contrast, the addition of β-catenin inhibitor KYA1797K attenuated the inhibitory effect of Akt activator SC-79 on PPARγ expression (Fig. 5d). These data suggest that activation of Akt stimulated GSK3β/β-catenin signaling, which in turn decreased the expression of PPARγ and inhibited adipogenesis of RCT-FAPs (Fig. 5e). To further confirm the role of β-catenin in regulating PPARγ and adipogenesis, β-catenin siRNA was used (Fig. 5f). Accordingly, the expression of PPARγ protein was increased by β-catenin knockdown (Fig. 5g). Increased adipogenesis of FAPs was also verified by oil red staining and RT-qPCR (Fig. 5h–j). These data demonstrate the importance of Akt/GSK-3ββ-catenin/PPARγ axis in mediating adipogenic differentiation of RCT-FAPs (Fig. 5e). In addition, when compared with ATT-FAPs, RCT-FAPs showed decreased Akt/GSK-3β/β-catenin signaling and upregulated PPARγ expression detected by western blotting (Fig. 5k). Taken together, it is likely that low activity of Akt/GSK-3β/β-catenin signaling in RCT-FAPs leads to upregulated PPARγ expression and contributes to increased adipogenesis ability.

### Activation of β-catenin alleviated fatty infiltration and improved shoulder function in RCT models

**Fig. 6:**
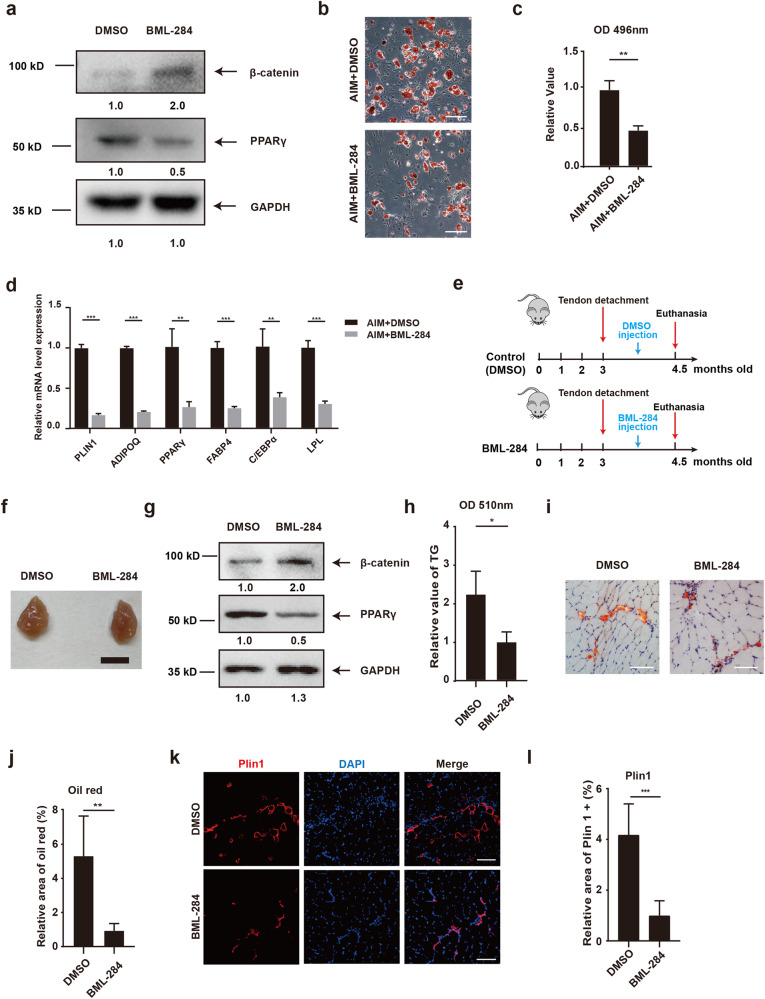
Activation of β-catenin alleviated fatty infiltration in RCT models. **a** Protein level of β-catenin, PPARγ, and GAPDH for RCT-FAPs (FAPs isolated from supraspinatus muscle one week after tendon tears) with or without β-catenin agonist BML-284 treatment. **b**, **c** The oil red staining and statistical analysis of RCT-FAPs after 7-day adipogenic induction in AIM with DMSO or BML-284 (*n* = 3). AIM adipogenic induction medium. Scale bar = 100 µm. **d** Relative mRNA expression level of adipogenic genes for RCT-FAPs after 7-day adipogenic induction in AIM with DMSO or BML-284 (*n* = 3). **e** Scheme of animal experimental design. **f** Gross appearance of supraspinatus muscles from the DMSO and BML-284 treatment groups. Scale bar = 50 mm. **g** The protein expression of PPARγ, β-catenin, and GAPDH of FAPs sorted from the DMSO group and BML-284 group. **h** Measurement of triglycerides in supraspinatus muscle between the BML-284 group and DMSO group (*n* = 4). **i**, **j** Oil red staining and statistical analysis of supraspinatus muscles from the DMSO group and BML-284 group (*n* = 5). Scale bar = 100 µm. **k**, **l** Immunofluorescence staining and statistical analysis of perilipin1 for supraspinatus muscles from the DMSO group and BML-284 group (*n* = 5). Scale bar = 100 µm. * indicated *p* < 0.05, ** indicated *p* < 0.01, and *** indicated *p* < 0.001.

Since our data showed reduced β-catenin activity was a key effector for increased adipogenesis of RCT-FAPs, we next investigated whether activation of β-catenin could alleviate fatty infiltration and improve shoulder function after rotator cuff injury. β-catenin activator BML-284 was used to treat RCT-FAPs in vitro. Western blotting results confirmed increased β-catenin activity and decreased PPARγ protein expression after BML-284 treatment (Fig. 6a). Oil red staining and RT-qPCR of adipogenic genes also showed that BML-284 could limit the adipogenic differentiation of FAPs in vitro (Fig. 6b–d). These data indicated BML-284 could be potentially used to inhibit muscle fatty infiltration after RCT.

To test this potential application, we conducted massive rotator cuff tears by complete transverse incision of supraspinatus and infraspinatus tendon in mice. The mice were randomly divided into two groups by SPSS software: BML-284 group received 50 ul 1 mM BML-284 injection in rotator cuff every 3.5 days for 6 weeks, while in the control group, muscular DMSO injection in rotator cuff muscle was performed with the same frequency (Fig. 6e). At the end of treatment, the supraspinatus muscles were harvested for lipid quantification analysis (Fig. 6f–i). Western blotting data from freshly isolated FAPs showed increased β-catenin and decreased PPARγ after treatment of BML-284 (Fig. 6g). Moreover, there were fewer triglycerides in supraspinatus muscle from BML-284 group than control group (Fig. 6h). Both oil red and immunofluorescence staining proved that fatty infiltration was significantly alleviated in the BML-284-treated supraspinatus muscle when compared to control group (Fig. 6i–l). Furthermore, the glycerol injury model was established as previous described [13], and BML-284 was used after injury. The immunofluorescence staining of Plin1 and triglyceride quantification revealed that the fatty infiltration of muscle after glycerol injury significantly decreased with treatment of BML-284 for two weeks (Fig. S1a–d), indicating the robust anti-adipogenic effect of β-catenin activator BML-284 in vivo.

**Fig. 7:**
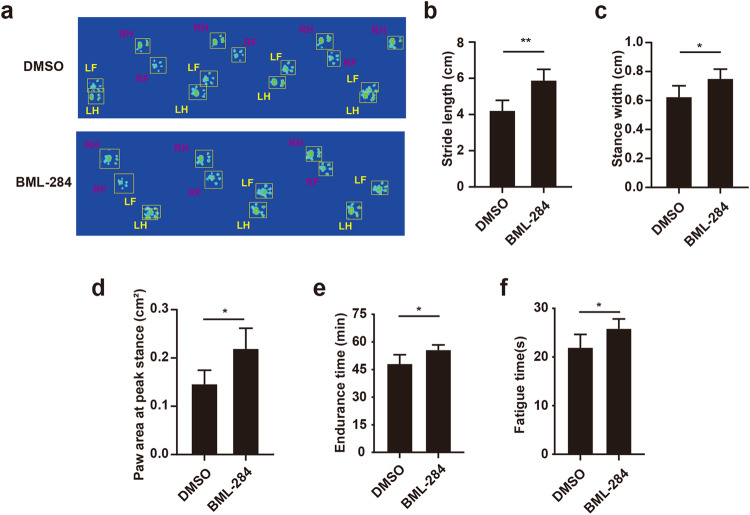
Activation of β-catenin promoted shoulder function and movement potential in RCT models. **a**–**d** Gait analysis of RCT mice with or without BML-284 treatment (*n* = 5). **e**, **f** Treadmill test of RCT mice with or without BML-284 treatment (*n* = 5). * indicated *p* < 0.05, ** indicated *p* < 0.01, and ns indicated *p* > 0.05.

Furthermore, to evaluate the shoulder function of RCT mice after BML-284 treatment, gait analysis and treadmill test were performed. Three parameters in gait analysis were recorded. The stride length represented shoulder abduction, the stance width indicated the ability to load the limb, and the paw area reflected the degree of chronic pain [20, 21]. Significant improvement of these parameters was revealed in RCT mice after BML-284 treatment, indicating the recovery of shoulder function (Fig. 7a–d). For treadmill test, longer endurance and fatigue time were observed in the BML-284 treatment group compared to the DMSO group (Fig. 7e, f). Thus, these data suggested that β-catenin activation holds therapeutic potential to abate excessive fatty infiltration of rotator cuff muscle and improve shoulder function after RCT.

## Discussion

In the current study, there was higher adipogenic differentiation potential in RCT-FAPs than ATT-FAPs. Suppressed Akt/GSK-3β/β-catenin signaling increased PPARγ expression and thus contributed to excessive adipogenesis in RCT-FAPs. Activation of Akt/GSK-3β/β-catenin signaling could be a therapeutic strategy to abate excessive fatty infiltration of rotator cuff muscle and improve function of shoulder joint after RCT.

As a common illness in orthopedic patients, rotator cuff tear always contributes to pain and functional limitations. Although surgical repair is routinely performed to treat patients with ruptured rotator cuff tendons, there is a high rate of tendon re-tearing after repair [5]. The fatty infiltration of the involved muscle is reported as a key factor affecting preoperative clinical manifestation and the surgical outcome after RCT [2, 5]. However, the in-depth mechanism underlying excessive fatty filtration after RCT remains unknown. Although muscular fatty infiltration frequently occurs after RCT, this pathology pattern is not ubiquitous in all musculotendinous injuries, especially for Achilles tendon tears [14, 15, 22]. This phenomenon indicates that there may exist distinct intrinsic differences among muscles from different anatomic locations [23, 24]. Since muscle fatty infiltration is mainly dependent on FAPs, it is plausible that the severe fatty infiltration in rotator cuff muscle could be due to the functional differences between FAPs residing in rotator cuff muscle and other muscles after tendon injury. However, there is a lack of literature exploring the knowledge in this field. Thus, the current study was performed.

One of the major findings of current study was that there was higher adipogenic differentiation potential in RCT-FAPs than ATT-FAPs. The distinct intrinsic differences of muscle tissues from different anatomical locations have been revealed [16, 17, 25]. However, the knowledge of differences of FAPs among different anatomic locations is limited, and even fewer studies focus on the FAPs from rotator cuff muscle. Since we believe both autonomous and environmental cues could influence the adipogenesis of FAPs, RCT and ATT models were firstly established. Then all the subsequent study was based on these tendon injury models. Thus, the finding in current study was more convincing to illustrate why fatty infiltration was more likely to occur in rotator cuff muscles after tendon injury.

The current study also indicated that suppressed Akt/GSK-3β/β-catenin signaling increased PPARγ expression and thus contributed to excessive adipogenesis in RCT-FAPs. Previous study has demonstrated the involvement of AKT and GSK-3β/β-catenin pathways in regulating the FAPs adipogenesis. Reggio et al. revealed that inactivation of AKT/mTOR pathways suppressed the adipogenic differentiation potential of FAPs in Duchenne muscular dystrophy mice [12]. In addition, it has also been illustrated that inhibition of GSK-3β could mitigate fatty infiltration in skeletal muscle after glycerol injury [13]. However, these studies were not based on tendon injury models, and the detailed underlying mechanism was also not explored. To clarify the mechanism underlying aberrant muscular fatty infiltration observed in RCT, we specifically employed Achilles tendon injury models as a control in this study. Since FAPs would undergo significant microenvironment changes including altered extracellular matrix composition, inflammatory mediator and growth factors, tendon injury model is the prerequisite to exploring the underlying mechanism behind variable muscular fatty infiltration. Through the comparison between RCT and ATT, our findings highlight and consolidate the crucial role of AKT and WNT pathways in determining adipogenesis of RCT-FAPs.

Since FAPs have emerged as a potential consequential cell source for muscular fatty infiltration [7], increasing investigators focused on modulating the number and activity of FAPs to address this pathology. Lemos et al. mitigated the fatty degeneration after RCT by activating TNF signaling and increasing FAPs apoptosis [26]. However, the early beneficial effects of FAPs for muscle milieu could be eclipsed by decreased number of FAPs [27]. Shirasawa et al. used imatinib to inhibit PDGFR signaling and found suppressed fatty infiltration in the RCT model [1]. While manipulating FAP-specific receptors may silence positive effects they may have, this strategy should be further investigated. In addition, Shirasawa et al. also demonstrated that retinoic acid receptor agonists suppressed adipogenesis of FAPs and thus muscle fatty infiltration could be alleviated [9], although the detailed mechanism remained further explored and there was a potential risk of muscle fibrosis progression using acid receptor agonists. Taken together, these studies proved that FAPs could be a potential target to abate fatty infiltration after RCT. The current study provided a treatment strategy for FAPs based on detailed cellular mechanisms underlying fatty infiltration after RCT. Since suppressed Akt/GSK-3β/β-catenin signaling contributed to excessive adipogenesis of RCT-FAPs, intramuscular injection of β-catenin activator BML-284 significantly attenuated fatty infiltration of rotator cuff muscles and rescued the shoulder movement function in the RCT models.

One of the primary limitations of present study was that the human RCT in clinical settings was a little bit different from mice RCT model in pathogenesis, age, and anatomy. Although mice RCT model could also yield consistent outcomes in muscular fatty infiltration, the results in current study should be transferred to humans with caution. In addition, although FAPs contributed a lot to fatty infiltration after RCT, the role of adipogenesis of other types of cells should not be ignored. Thus, β-catenin agonist BML-284 might alleviate fatty infiltration by targeting other involved cell populations. Furthermore, the RCT-FAPs and ATT-FAPs investigated in current study were obtained only from one time point (one week after tendon injury). The time-dependent changes of FAPs after tendon injury should be further investigated. All issues above need to be fully addressed to thoroughly clarify the mechanism and treatment of excessive muscular fatty infiltration after RCT.

In conclusion, there was higher adipogenic differentiation potential in RCT-FAPs than ATT-FAPs. Suppressed Akt/GSK-3β/β-catenin signaling increased PPARγ expression and thus contributed to excessive adipogenesis in RCT-FAPs. Modulation of Akt/GSK-3β/β-catenin signaling ameliorated excessive fatty infiltration of rotator cuff muscles and improved shoulder function after RCT.

## Materials and methods

### Animal

Male C57BL/6 mice (twelve-week-old) were purchased from Animal Model Research Center of Nanjing University. The RCT and ATT animal models were established as previously described [28–30]. Briefly, a complete transverse incision of Achilles tendon approximately 3 mm from the calcaneus was performed for ATT animal model, and a complete transverse incision of supraspinatus and infraspinatus tendons near tendon insertion was performed for RCT animal model. The experiments were approved by ethical committee in local institution (Approval NO. XHEC-F-2022-064).

### Muscle harvest and fluorescence-activated cell sorting

Supraspinatus, infraspinatus, and gastrocnemius muscles were dissected one week after tendon injury. The muscles were cut into pasty and digested by collagenase II (Worthington biochemical, 700–800 U/ml, cat#LS004177) for 45 min. Then they were further digested with the mixtures of dispase (Life Technologies, 11 U/ml, cat#17105041) and collagenase II for 30 min. The single-cell suspension was stained with APC-CD31 (Biolegend, cat#102510), APC-CD45 (Biolegend, cat#103112), PE-PDGFRα (Thermo Fisher Scientific, cat#12-1401-81) for 40 min at 4 °C. Finally, CD45-CD31-PDGFRα+ murine FAPs were obtained by BD Influx sorter (BD Biosciences) [31].

### Cell culture, treatment, and adipogenic differentiation

The FAPs were cultured in growth medium on Matrigel-coated (Biocoat, cat#354277) plates. The growth medium contains high-glucose DMEM (Gibco, cat#12100-046), 20% FBS (Gibco, cat#10-013-CV), 2.5 ng/ml bFGF (R&D systems, cat#233-FB-025), and 1% Penicillin-Streptomycin (Gibco, cat#15140-122). 10 μM SC-79 (Beyotime, cat#SF2730), 1 μM BML-284 (Medchemexpress, cat#HY-19987) and 1 μM KYA1797K (Medchemexpress, cat#HY-101090) were applied during the specific experiment. Adipogenic induction medium (AIM) was used to induce the adipogenic differentiation for 7 days [32]. The AIM contains 20% FBS, 1 μg/mL insulin (Sigma-Aldrich, cat#I2643), 0.25 µM dexamethasone (Sigma-Aldrich, cat#D4902), 0.5 mM 3-isobutyl-1-methylxanthine (Sigma-Aldrich, cat#I5879), and 1% penicillin-streptomycin.

### Immunohistology and Immunofluorescent staining

Cultured cells or frozen tissue slices were fixed in 4% paraformaldehyde for 20 min, blocked with 1% BSA for 1 h and permeabilized by 0.5% Triton X-100 for 30 min. Then they were incubated with anti-PDGFRα (Abcam, cat#ab203491), anti-Laminin (Abcam, cat#ab44941), and anti-perilipin A/B (Millipore, cat#P1873) at 4 °C overnight. Subsequently, Alexa 488- or Alexa 594-labeled anti-mouse or rabbit secondary antibodies (Invitrogen) were used at room temperature for 1 h. The nuclei were stained by 4, 6-diamidino-2-phenylindole (Sigma-Aldrich, cat#D9542) for 5 min. The images were acquired by BX53 or IX73 fluorescence microscope (Olympus). All the images were analyzed by image J or image-pro plus 6.0 software.

### Oil red staining and triglycerides quantification analysis

Tissue slices or cultured cells were first fixed in 4% paraformaldehyde for 10 min, followed by oil red (Solarbio, cat#G1260) treatment for 10 min. Then samples were stained with hematoxylin for 5 min and mounted with 10% glycerol. For quantification analysis of lipid after adipogenic differentiation, the oil red was extracted by isopropanol for 5 min, and the value at 496 nm absorbance was detected by a microplate reader (Thermo Fisher Scientific).

Triglycerides colorimetric assay kit (Elabscience, cat#E-BC-K261-M) was employed to quantify the muscular fatty infiltration after tendon injury. Briefly, the muscle was first grounded with isopropanol (ACMEC, cat#I86620). Then tissue fragments were discarded by centrifugal machine at 10000 × *g* for 3 min. The supernatant was used to detect triglycerides by microplate reader at 510 nm absorbance.

### Gait analysis

Noldus Catwalk system was administrated to perform gait analysis after RCT. Weights were measured to avoid interference of body size. All mice walked through the tunnel of catwalk system and then the stride length, stance width, stance time, and paw area at the peak stance were recorded [33, 34].

### Treadmill test

The treadmill test was performed by treadmill apparatus (ZII-PT/5 S). In endurance test, mice run on the treadmill at a constant speed of 20 m/min. In exhaustion test, the speed began at 16 m/min and was increased 2 m/min per 3 min. The situation that mice were incapable of running for 10 s would be identified as exhaustion.

### Western blotting

Lysed proteins with loading buffer were separated and transferred to nitrocellulose membranes according to the standard methods. Full membranes were blocked by 5% nonfat milk in TBST for 1 h at room temperature. Primary antibodies were incubated overnight at 4 °C: anti-GAPDH (CST, cat#2118 L), anti-Akt (CST, cat#4865), anti-phospho-Akt (CST, cat#4060), anti-GSK-3β (Santa Cruz, cat#sc-53931), anti-phospho-GSK-3β (CST, cat#93336), anti-β-catenin (CST, cat#8480), anti-PPARγ (CST, cat#2435). Then the sample was incubated by HRP-conjugated secondary anti-rabbit or anti-mouse IgG antibodies (Ray antibody biotech, cat#RM3004) for one hour at room temperature. Lumi Q ECL luminescent liquid was used to capture the target strips.

### Transfection of siRNA

In total, 2 μl lipofectamine 2000 (Invitrogen, cat#11668019) was mixed with 200 μl Opti-MEM™ I reduced serum medium (Thermo Fisher, cat#31985062) and 1 μl 100 μM β-catenin siRNA at room temperature for 20 min. The mixture was then equably added into the 12-well plate cultured with FAPs. The sequence of siRNA was listed as follow: β-catenin siRNA 5ʹ-GCAGAATACAAATGATGTA-3ʹ.

### Gene expression analysis

Total RNA was extracted by EZ-press RNA Purification Kit (EZ Bioscience, cat#B0004DP) according to manufacturer’s instruction. RNA quantification was measured by NanoDrop 2000 (Thermo Fisher Scientific). One microgram total RNA was reverse transcribed into cDNA with M-MuLV Reverse Transcriptase (NEB, cat#M0253L). The products of reverse transcription were used for Real-Time qPCR. Quantitative reverse transcription PCR was performed by FastStart Universal SYBR Green Master (Roche, cat#4913914001) in the ABI Quant Studio 6 real-time qPCR system. The primers of RT-qPCR were shown in Table 1.

**Table 1:** Primer sequences for real-time PCR.

Target Gene	Sequences
Mouse -LPL-F	5ʹ-TTGCCCTAAGGACCCCTGAA-3ʹ
Mouse -LPL-R	5ʹ-TTGAAGTGGCAGTTAGACACAG-3ʹ
Mouse -C/EBPα-F	5ʹ-GCGGGAACGCAACAACATC-3ʹ
Mouse -C/EBPα-R	5ʹ-GTCACTGGTCAACTCCAGCAC-3ʹ
Mouse -Perilipin 1-F	5ʹ-CTGTGTGCAATGCCTATGAGA-3ʹ
Mouse -Perilipin 1-R	5’-CTGGAGGGTATTGAAGAGCCG-3'
Mouse-GAPDH-F	5ʹ-AGGTCGGTGTGAACGGATTTG-3ʹ
Mouse-GAPDH-R	5ʹ-GGGGTCGTTGATGGCAACA-3ʹ
Mouse -ADIPOQ-F	5ʹ-GAAGCCGCTTATGTGTATCGC-3ʹ
Mouse -ADIPOQ-R	5ʹ-GAATGGGTACATTGGGAACAGT-3ʹ
Mouse -PPAR γ-F	5ʹ-GGAAGACCACTCGCATTCCTT-3ʹ
Mouse -PPAR γ-R	5ʹ-GTAATCAGCAACCATTGGGTCA-3ʹ
Mouse -FABP4-F	5ʹ-AAGGTGAAGAGCATCATAACCCT-3ʹ
Mouse -FABP4-R	5ʹ-TCACGCCTTTCATAACACATTCC-3ʹ

### Bulk RNA-seq and analysis

RNA sequencing libraries were obtained by NEBNext Ultra RNA Library Prep Kit for Illumina (NEB, cat#E7530L). The paired-end RNA-seq sequencing library was sequenced by the Illumina NovaSeq 6000 sequencer (2×150 bp read length). The raw paired-end reads were embellished and quality controlled by fastp (https://github.com/OpenGene/fastp) with default parameters [35]. Differential expression analysis with P-adjust ≤ 0.001 (DEGseq) were considered significantly different expressed genes. GO and KEGG analyses were performed by Goatools (https://github.com/tanghaibao/Goatools) and KOBAS (http://kobas.cbi.pku.edu.cn/home.do) [36].

### Statistical analysis

Statical analysis was conducted by GraphPad Prism 8 (San Diego, USA) software. Student t test was used to compare quantitative parameters between two groups. The histological evaluation was used as primary outcome. The equation, *n* = 2(Z_α/2_ + Z_β_)^2^σ^2^/(μ_c_-μ_t_)^2^, was used to calculate sample size [37], and the alpha value was set as 0.05 with a power of 0.80. *P* < 0.05 was considered statistically significant. All the experiment results were presented by mean ± standard deviation. Technical replicates or biological repeats were introduced in the figure legend. All the experiments were assessed and analyzed by two independent investigators (H.Z. and X.S.) in a blinded manner.

### Supplementary information

Supplemental Figure Legend of sFigure1

Figure S1

## Data Availability

The datasets generated and/or analyzed in the study are available from corresponding author on reasonable request.
