# Supplementary material for: Suppressed Akt/GSK-3β/β-catenin signaling contributes to excessive adipogenesis of fibro-adipogenic progenitors after rotator cuff tears
Source: Cell Death Discov. 2023 Aug 25;9:312. doi: 10.1038/s41420-023-01618-4 (PMC10457376; doi:10.1038/s41420-023-01618-4)
Supplement: Supplementary file 1 — Supplemental Figure Legend of sFigure1 [file 41420_2023_1618_MOESM1_ESM.docx]

**Figure S1** Activation of β-catenin alleviated fatty infiltration in glycerol injury models.

(a) Scheme of animal experimental design.

(b) Measurement of triglycerides in gastrocnemius muscle between the BML-284 group and DMSO group (n=5).

(c) and (d) Immunofluorescence staining and statistical analysis of perilipin1 for gastrocnemius muscles from the DMSO group and BML-284 group (n=6). Scale bar = 100 µm.
