# Supplementary figures and images for: Suppressed Akt/GSK-3β/β-catenin signaling contributes to excessive adipogenesis of fibro-adipogenic progenitors after rotator cuff tears
Source: Cell Death Discov. 2023 Aug 25;9:312. doi: 10.1038/s41420-023-01618-4 (PMC10457376; doi:10.1038/s41420-023-01618-4)

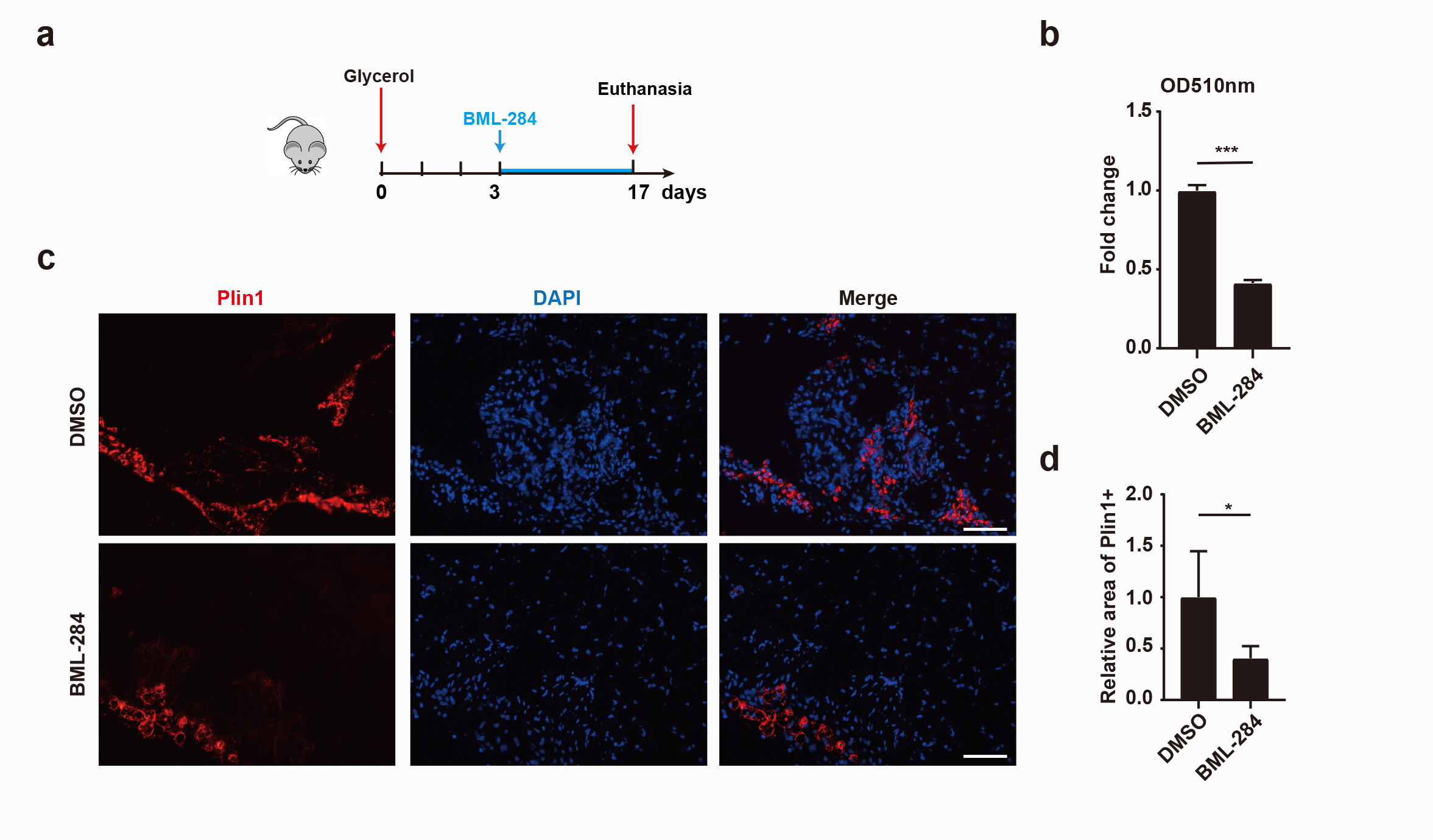

Supplement: Supplementary file 2 — Figure S1 [file 41420_2023_1618_MOESM2_ESM.png]
